# Supplementary material for: Millisecond dynamics of BTK reveal kinome-wide conformational plasticity within the apo kinase domain
Source: Sci Rep. 2017 Nov 15;7:15604. doi: 10.1038/s41598-017-10697-0 (PMC5688120; doi:10.1038/s41598-017-10697-0)
Supplement: Supplementary file 1 — Supporting Information [file 41598_2017_10697_MOESM1_ESM.pdf]

Supplementary Information

Millisecond dynamics of BTK reveal kinome-wide conformational plasticity within the apo kinase domain

Mohammad M. Sultan[1], Rajiah Aldrin Denny[2#], Ray Unwalla[2], Frank Lovering[2], & Vijay S. Pande[1†]

[1]Department of Chemistry, Stanford University, 318 Campus Drive, Stanford, California 94305, USA.

[2]Medicine Design, Worldwide Research & Development, Pfizer Inc, 610 Main St, Cambridge, MA 02139, USA.
[†]Pande@stanford.edu
[#]Aldrin.Denny@pfizer.com

Complete Methods:

Initial Setup and homology modeling:

We downloaded all 23 publically available BTK pdbs from the protein databank[1]. We then used Modeller[2] to mutate out all the sequences to the human sequence (Uniprot ID Q06187) while building in missing loops and residues. We also used Modeller to mutate ASP539 to its protonated form ASH. Hydrogen atoms were added to both the deprotonated and protonated structures using the t-leap module contained with the Amber tools suite[3–5]. All the structures were solvated in a water box with 10 Å padding on all sides. Chloride and sodium ions were added to neutralize the charge and set up the final ionic concentration to 150mM. The Amber99sb-ildn[6] force field was used to model protein dynamics in conjunction with the TIP3P[7] water model. This process led to the creation of 23 starting crystallographic structures for each of the two systems.

Minimization:

We minimized the system in two steps using Amber. In the first step, the protein was held fixed with 500 kcal/mol restraints while the water and counter ions were minimized for 1,000 steps using steepest gradient descent followed by 3,000 steps of conjugate gradient descent. We then released the restraints on the proteins and minimized the entire system for 2,000 steps using steepest gradient descent followed by another 2,000 steps of conjugate gradient descent. We then loaded the minimized Amber topology and input coordinates into OpenMM[8] for simulation setup and equilibration.

Equilibration:

All production runs for regular molecular dynamics simulations were run at a pressure of 1 atm and temperature of 300 K. The Langevin integrator with a friction coefficient of 1/ps and a 2 fs time step were used. A Monte Carlo barostat with an interval of 25 frames was used to maintain the pressure at 1 atm. Frames were saved every 200 ps. Long-range electrostatics were dealt with using the Particle mesh Ewald[9] algorithm with a 10 Å cutoff for the production runs on Folding@home[10]. Hydrogen bonds were constrained using the LINCS[11] algorithm for the Folding@home production runs. All simulations were equilibrated for 1 ns before beginning production runs.

Production MD Simulations:

For each of the 46 starting configurations, we started 35 simulations with randomized velocities sampled from a Boltzmann distribution. We also performed a round of adaptive sampling where we reseeded simulations from configurations sampled along high free energy paths for both systems. Overall, we collected 887 μs (1,065 trajectories with 395 ns median length) for the BTK-ASP simulations and 818 μs (1,075 trajectories with 400 ns median length) for BTK-ASH simulations. We then built a joint Markov model for the entire 1.7 ms of data. The supporting information contains more statistics for the individual production simulations.

Markov state model:

Building a MSM requires identification of metastable kinetically similar states. This splitting of the phase space is followed by counting the transitions between those states as observed in our

trajectories at a Markovian (memory free) lag time. This means that the probability of moving to the next state only depends upon the current state and nothing before it. The conditional independence means that we only require local equilibrium[12] within the state to be able to infer global properties, allowing us to parallelize sampling across hundreds of machines. This transition model can be summarized using the following equation:

$$p(t+\tau) = p(t)T(\tau) \ (1)$$

where $p(t)$ is the probability distribution at time "t" while $p(t+\tau)$ is the probability distribution after a Markovian lagtime τ. Spectral decomposition of the MSM transition matrix was used to estimate the equilibrium populations and dynamical processes connecting those Markov states. The relaxation timescales for these dynamical processes can be obtained by using the following transformation on the associated eigenvalue μ

$$Relaxation\ timescale = -\frac{\tau}{\ln(\mu)} \ (2)$$

The MSM framework avoids the collective variable problem of many enhanced sampling algorithms by only using unbiased simulations in the full state space of the protein. We note that all of the trajectories used or presented in the paper were obtained via regular MD sampling. While, this means that MSMs generally require more unbiased sampling, it has the advantage of NOT specifying CVs or having to worry about hidden degrees of freedom. In fact, thanks to the tICA[13] algorithm, our simulations tell(SI Figure 6-7) us what the important degrees of freedom are. The trick to getting MSM to work is to start from as many available crystal structures and to re-seed simulations from lowly populated states, both of which were employed in this paper.

After sampling the MD trajectories using Folding@home, a total of 2,140 trajectories were vectorized using the protein dihedrals and closest contacts distances. While previous modeling work on kinases with MSMs[14] used RMSD-based metrics, recent advances in dimensionality reduction techniques[13,15] now allow us to learn a kinetically relevant structure-based distance metric directly from the MD trajectories. We used the sine and cosine values of the χ_1 dihedrals for the protein. The trigonometric transform was necessary to account to dihedral periodicity within our algorithms. For the contacts, we used the closest heavy atom distance between all N-choose-2 residues from the following list of residues : GLY393, TRP395, GLU396, ILE397, PRO399, LEU402, LEU405, LEU408, GLY411, GLY414, LYS417, LYS420, GLY423, ASP426, ILE429, LYS430, ILE432, GLY435, SER438, GLU441, GLU444, GLU445, LYS447, MET450, SER453, LYS456, GLN459, GLY462, THR465, ARG468, PHE471, THR474, MET477, GLY480, LEU483, LEU486, MET489, ARG492, THR495, LEU498, MET501, ASP504, GLU507, GLU510, GLU513, GLN516, HIS519, ARG520, ASP521, LEU522, ARG525, LEU528, ASP531, VAL534, VAL537, ASP539, PHE540, GLY541, SER543, ARG544, VAL546, ASP549, TYR551, THR552, VAL555, LYS558, PHE559, PRO560, VAL561, SER564, GLU567, MET570, LYS573, SER576, ASP579, ALA582, VAL585, TRP588, TYR591, GLY594, PRO597, ARG600, ASN603, THR606, HIS609, GLN612, ARG615, ARG618, LEU621, GLU624, TYR627, MET630, CYS633, GLU636, ASP639, PRO642, LYS645, LEU648, ILE651, VAL654, GLU657. This list was designed to explicitly include the following conserved kinase core residues Trp395, Glu396, Ile397, Lys430, Glu445, Asp539, Phe540, Gly541, His519, Arg520, Asp521, Arg544, Tyr551, Phe559, Pro560, and Val561. This subsampling was necessary to reduce the number of

contacts to a computationally manageable subset. This feature selection led to each frame being represented as a feature vector of length 5,532. We preprocessed the data by setting the mean of each feature to 0 and variance to 1. We used the same feature selection scheme for both the BTK_{ASP} and the BTK_{ASH} datasets. We then reduced the dimensionality of the datasets using time structure independent component analysis (tICA)[13,15]. tICA seeks to find a set of linear combinations of features that de-correlate the slowest (at a certain lag time) while minimizing their correlation. This is done by solving the following generalized eigenvalue problem:

$$C(\tau)\mathbf{v} = \boldsymbol{\lambda}\Sigma\mathbf{v} \quad (3)$$

where $\mathbf{v}$ are the associated eigevectors (tICs), $\boldsymbol{\lambda}$ the eigenvalues, and Σ is the covariance matrix

$$\Sigma_{ij} = \mathrm{E}[\mathbf{X}_{\mathrm{i}}(t)\mathbf{X}_j(t)] \quad (4)$$

and $C(\tau)$ is the time lagged correlation matrix whose ij element is defined as

$$C(\tau)_{ij} = \mathrm{E}\big[\mathbf{X}_{\mathrm{i}}(t)\mathbf{X}_j(t+\tau)\big] \quad (5)$$

In equations 4 and 5, $\mathrm{E}[\ldots]$ is the average/expectation over the entire ensemble. The aim of equation 3 is to find the slowest/most highly auto-correlated set of coordinates ($\mathbf{v}$) with in our dataset at a certain lag time. Here, τ is the tICA lagtime and can be different from Markovian lagtime. The tICA-transformed dataset was clustered using the K-means algorithm. We then used the cluster labeled dataset to build a MSM.

It is worth noting that we built a single tICA and K-Means model for the deprotonated and protonated ensembles. Given the state-space equivalence, this allows us to explicitly compare the thermodynamics and kinetics of these systems. We also note for all projections, the deprotonated (BTK_{ASP}) ensemble's highest populated state was assigned an absolute free energy of 0 kcal/mol and all other free energies were reported relative to that state. Based upon previous work[14,16] and the convergence of the implied timescales plot (Supporting figure 1) for 50-500 state models, we chose a Markovian lag time of 80 ns. For all the other hyper-parameters within the model, we turned to cross validation.

Hyperparameter cross validation:

Recently McGibbon et al.[17,18] formulated a cross-validation scheme for the selection of hyperparameters for Markov state models. The methodology requires maximization of the generalized matrix Rayleigh quotient (GMRQ) across training and testing sets. The maximization of the test set GMRQ leads to the finding of a rank-m projection that best captures the slow dynamics of the system. For Markov models, this has two tunable parameters: the value of m and the Markovian lag time. Based upon previous work and initial modeling, these were set to 5 and 80 ns, respectively. In order to quickly fit models, the trajectories were subsampled to every 8 ns. The GMRQ was used to optimize over the tICA lag time (8 ns-4 μs), number of tICA components (between 1 and 10), choice of kinetic mapping[19] (yes/no) and number of cluster centers (50-500). We used 5-fold shuffle split with a test size of half the data to ensure that the method doesn't overfit to the training set. After randomly sampling several thousand models from this hyperparameter space, we picked the highest scoring model with GMRQ score of 5.85 (out of a theoretical maximum of 6 with an unknown true upper bound). The parameters for the best model are given below:

Hyper parameter	Value in best model
tICA lagtime	208 ns
tICA number of components	3
tICA kinetic mapping	True
Number of clusters	190

Table 1: GMRQ scored hyper parameters for the best model.

After we determined the optimal model given the current amount of sampling, we retrained the model on the entire set of trajectories. For the reported tICA model, we used a sparse variant of tICA[20] for increased interpretability (Supporting Figure 6-7).

The Markov transition matrix was fit via maximum likelihood estimation (MLE) with reversibility and ergodicity constraints. This procedure discarded no data for both ensembles, indicating converged sampling. To obtain error bars for the equilibrium populations, 200 rounds of bootstrapping were performed over the original set of trajectories. We performed standard bootstrapping (N=200) where for each bootstrap sample, we randomly sampled T (T=1065 for BTK-ASP, 1075 for BTK-ASH) trajectories, *with re-replacement*, from our ensemble. We explicitly kept the final micro-state definitions to allow for direct comparisons. We then fit a new MSM to this perturbed dataset. Due to this sampling-with-replacement, each bootstrap sample produced a slightly different MSM and thus different estimates for both the thermodynamics and kinetics, allowing us to measure the uncertainty within our model (Supporting Figure 2). All populations and free energies reported are from the MLE populations with the 95% confidence interval for the free energies coming from the 200 rounds of bootstrap sampling. The bootstrapping was used to find empirical estimates for the standard deviations of equilibrium population of a state which were then used to obtain the upper and lower bound for the populations using

$$lower\ population\ bound, upper\ population\ bound = \left(\bar{x} - 1.96\frac{\sigma}{\sqrt{200}}, \bar{x} + 1.96\frac{\sigma}{\sqrt{200}}\right) \quad (6)$$

where $\bar{x}$ is the MLE estimate for state's population while σ is the boostrap estimate for the standard deviation of the population. Repeating this analysis for all 190 states gives us the error estimate in the population, and thus the error in the estimated free energy, for each of those states. The mean and errors can then be propagated into projections (Supporting figure 12) for further analysis.

The trajectories were featurized and analyzed using the MDTraj[21] package while tICA dimensionality reduction and Markov modeling were performed using MSMBuilder[22]. Most of the analysis was performed within the IPython/Jupyter scientific environment[23] with extensive use of the matplotlib[24], and scikit-learn libraries[25]. All protein images were generated using visual molecular dynamics (VMD)[26], all protein surfaces were rendered using SURF[27],and secondary structure was assigned using STRIDE[28] as implemented in VMD.

Model interpretation:

The models were primarily analyzed using techniques laid out in previous papers[15,29]. To further query the model, we sampled a 800 μs long kinetic Monte Carlo trajectory (10,000 frames at a lagtime of 80 ns) from the Markovian transition matrix.

Starting structures:
We started simulations from the following set of coordinates.
3GEN 3K54 3PIX 3PIZ 3PJ1 3PJ2 3PJ3 4RFZ 4RFY 4RG0 3P08 3OCS 1K2P 4OTF 3OCT 4NWM 4XI2 4Y93 4Y95 4OT5 4OTQ 4OTR 4OT6.
All of the pdbs were mutated to the following sequences using Modeller[2].
BTK-ASP:
GSWEIDPKDLTFLKELGTGQFGVVKYGKWRGQYDVAIKMIKEGSMSEDEFIEEAKVMM
NLSHEKLVQLYGVCTKQRPIFIITEYMANGCLLNYLREMRHRFQTQQLLEMCKDVCEA
MEYLESKQFLHRDLAARNCLVNDQGVVKVSDFGLSRYVLDDEYTSSVGSKFPVRWSPP
EVLMYSKFSSKSDIWAFGVLMWEIYSLGKMPYERFTNSETAEHIAQGLRLYRPHLASEK
VYTIMYSCWHEKADERPTFKILLSNILDVMDEES
BTK-ASH:
GSWEIDPKDLTFLKELGTGQFGVVKYGKWRGQYDVAIKMIKEGSMSEDEFIEEAKVMM
NLSHEKLVQLYGVCTKQRPIFIITEYMANGCLLNYLREMRHRFQTQQLLEMCKDVCEA
MEYLESKQFLHRDLAARNCLVNDQGVVKVS**D**$^{+}$FGLSRYVLDDEYTSSVGSKFPVRWSP
PEVLMYSKFSSKSDIWAFGVLMWEIYSLGKMPYERFTNSETAEHIAQGLRLYRPHLASE
KVYTIMYSCWHEKADERPTFKILLSNILDVMDEES

Structural definitions:

State	**C-helix**	**A-loop**	**DFG**
**Active**	In	Unfolded	In
**Src-like**	Out	Folded	In
**DFG^{out}**	Either	Either	Out
**Intermediate**	Out	Either	In

Table 2: Summary of motif positioning for active, Src-like and DFG^{out} kinase states. All, except the active, states are catalytically inactive. See Figure 8 below for more details.

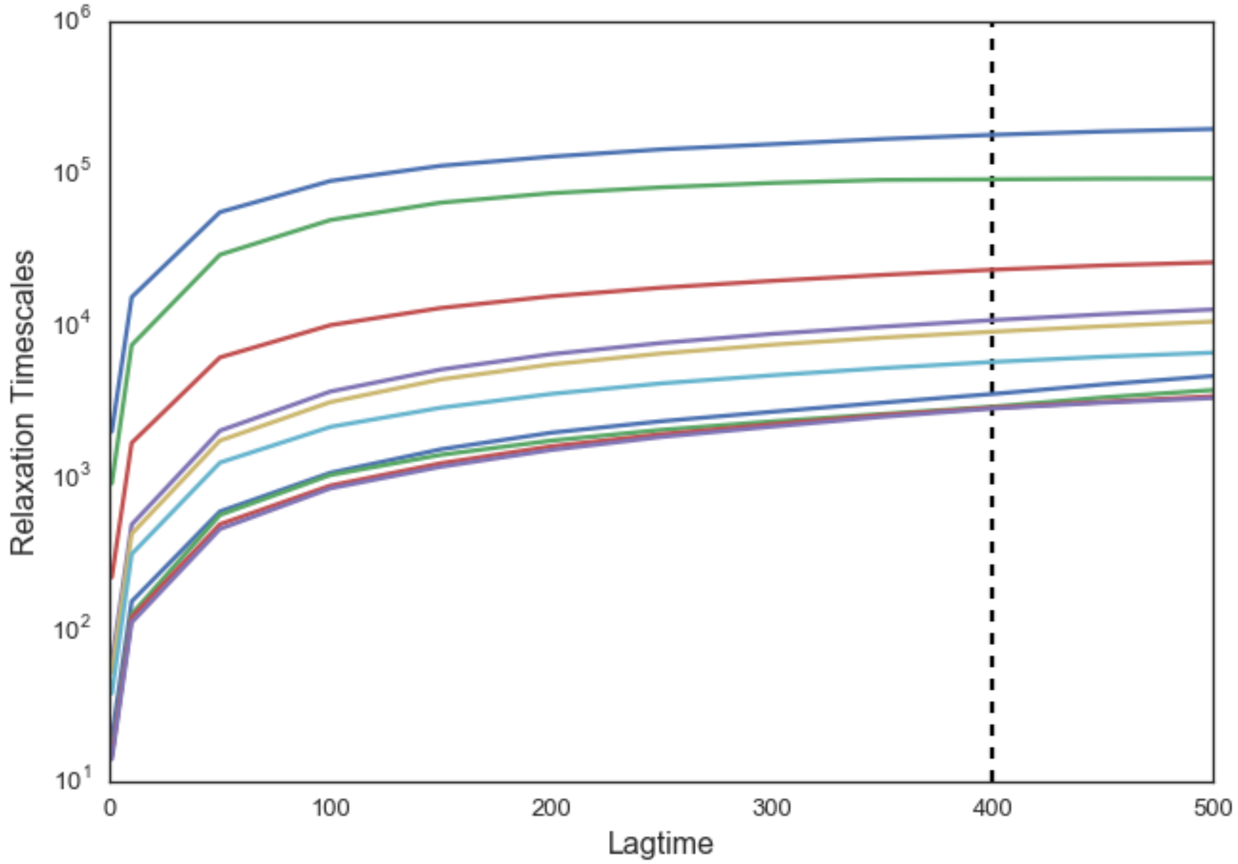

*Figure 1:Validation of the Markovian lagtime for the BTK-ASP model. The logrithmic convergence of longest relaxation timescales implies that the model kinetics are independent of the chosen lagtime. The trajectory frames were stored every 200ps which means a Markovian lagtime of 400*200=80ns(vertical dotted line). The longest deactivation relaxtation timescale has an associated value ~200ps*10**5=~20-100microseconds.*

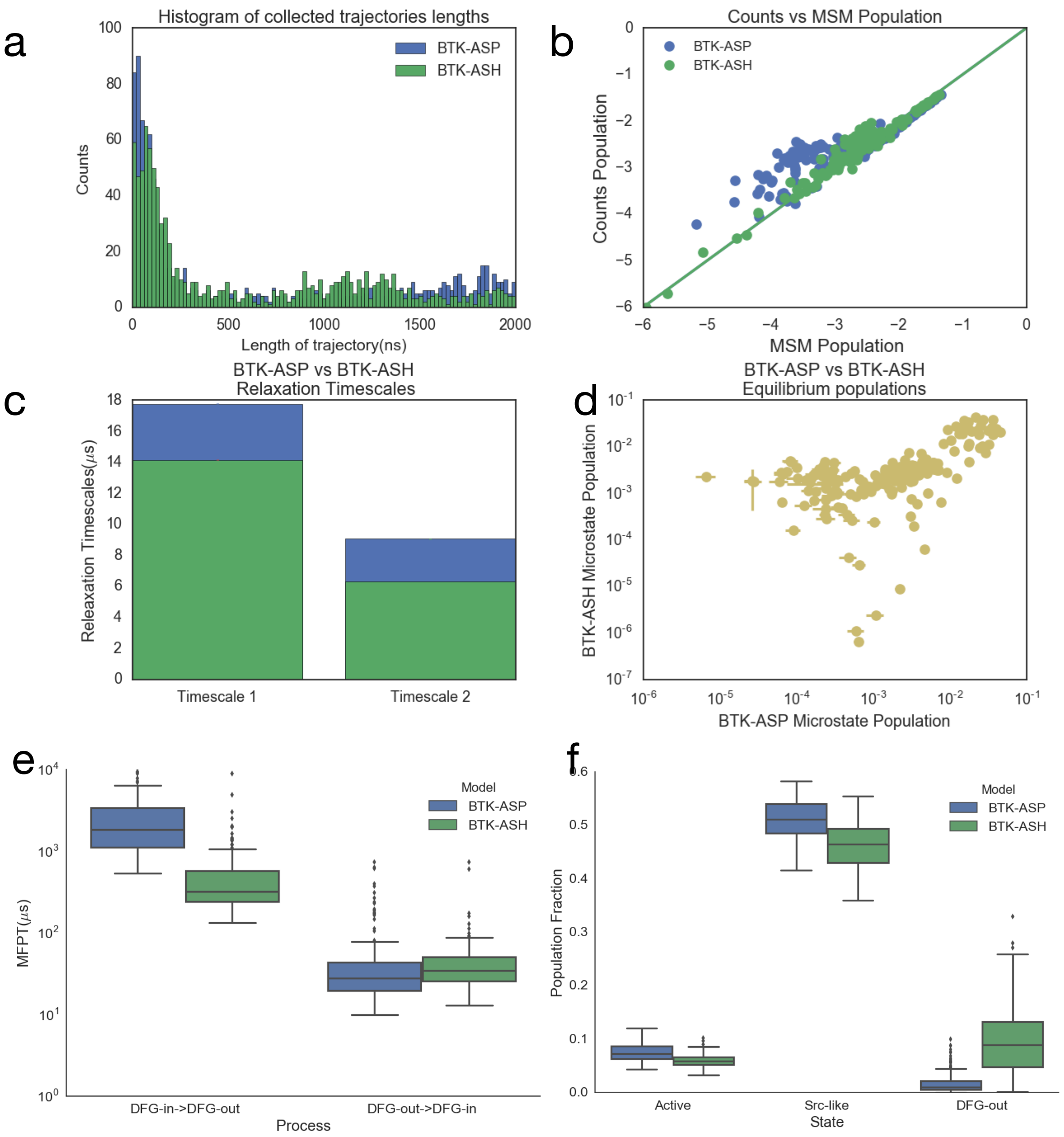

Figure 2: Statistical validation of the reporeted MSM. Topwise from left. a). Histogram of collected trajectory lenghts for both the BTK-ASP and BTK-ASH model. The median trajectrory length for both datasets was 395- 400ns and the aggregate simulation time was 800-900µs apiece. b). Predicted MSM model population versus the raw counts plotted on a log scale. c). The longest timescales for both the BTK-ASP and BTK-ASH models are in the 10s of µs. For both models, the longest timescales correspond to the DFG flip and deactivation. d). Equilibritum population and associated S.E.M for both models. e). Distribtuon of the median values for DFG transition times across all bootstrap models. f).Distributon of macrostate populations across all bootstrapped models. The error bars were obtained using 200 rounds of bootstrap sampling as outlined in Methods.

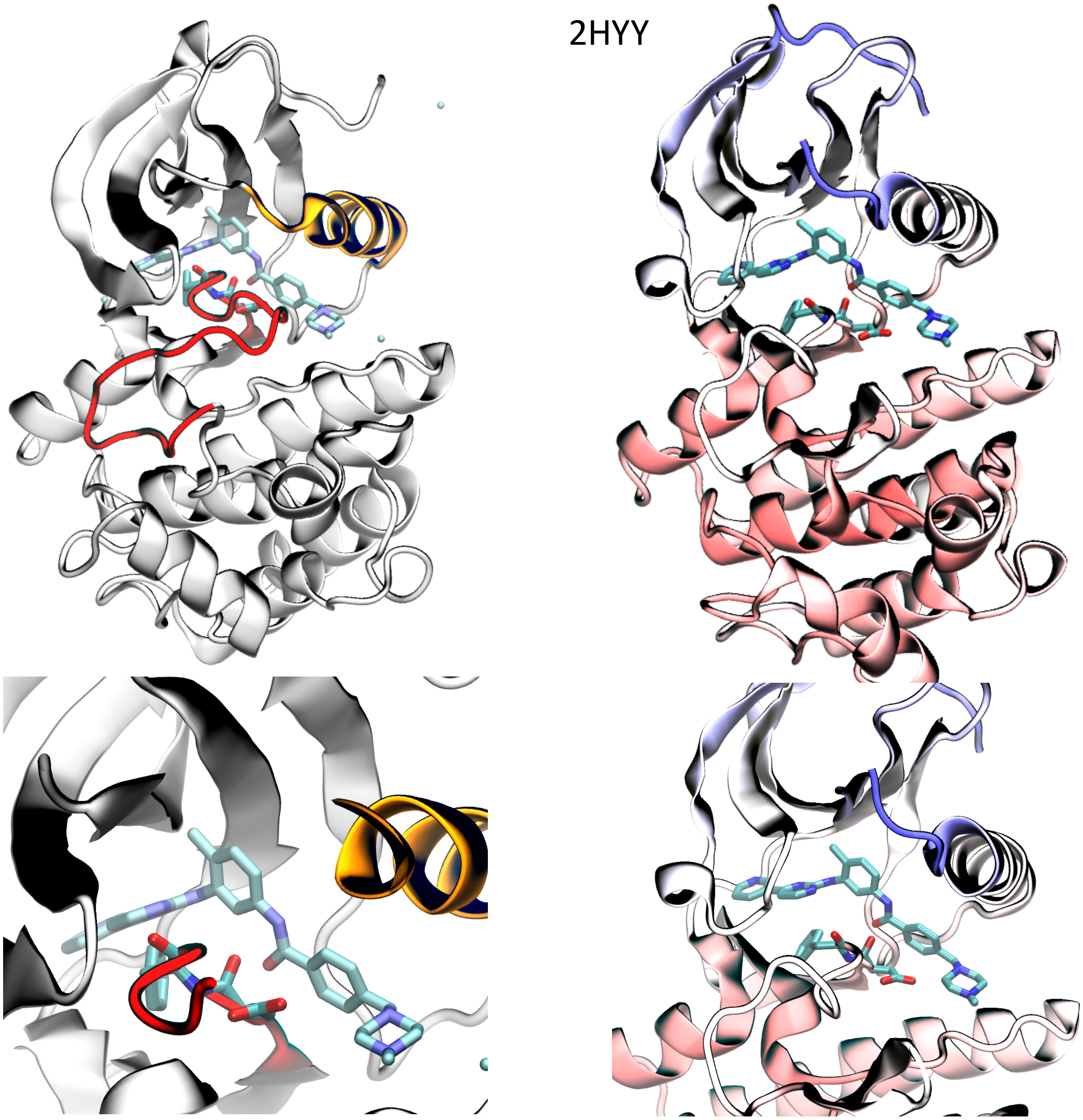

Figure 3: Comparison of a randomly sampled predicted DFG-out(type 2) state for BTK vs a prototypical DFG-out(type 2) drug bound kinase, Abl(2HYY). The coloring for Abl corrsponds to the beta values as reported in the crystal structures. The coloring for BTK, follows the conventions reported in the main text.

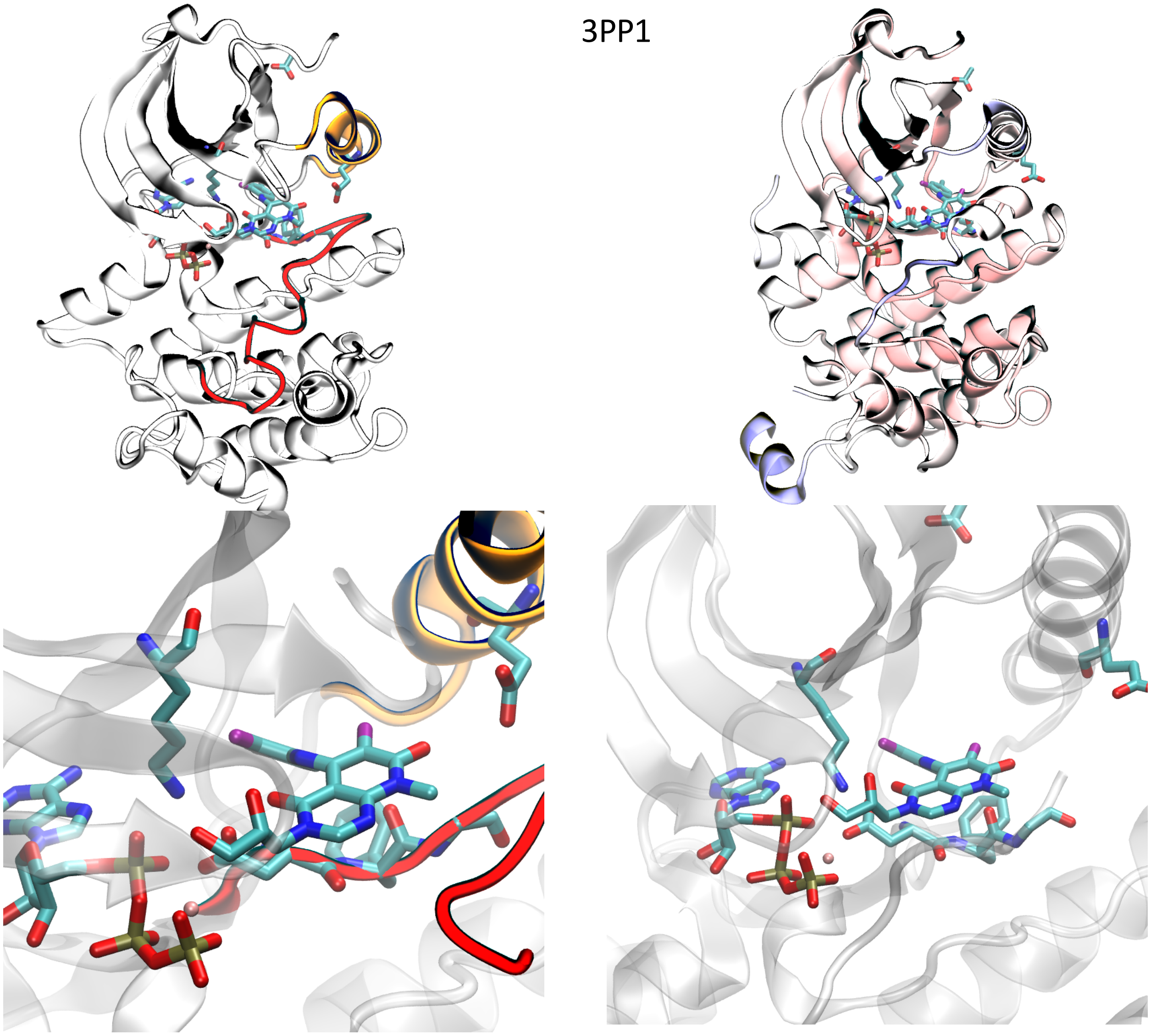

Figure 4: Comparison of a randomly sampled predicted FG-down-C-helix out state for BTK vs a FG-down-C-helix out state Mek kinase(3PP1). The coloring for Mek corrsponds to the beta values as reported in the crystal structures. The coloring for BTK, follows the conventions reported in the main text. The outward rotation of the C-helix opens up a allosteric pocket that can potentially be used to design allosteric inhibitors of BTK.

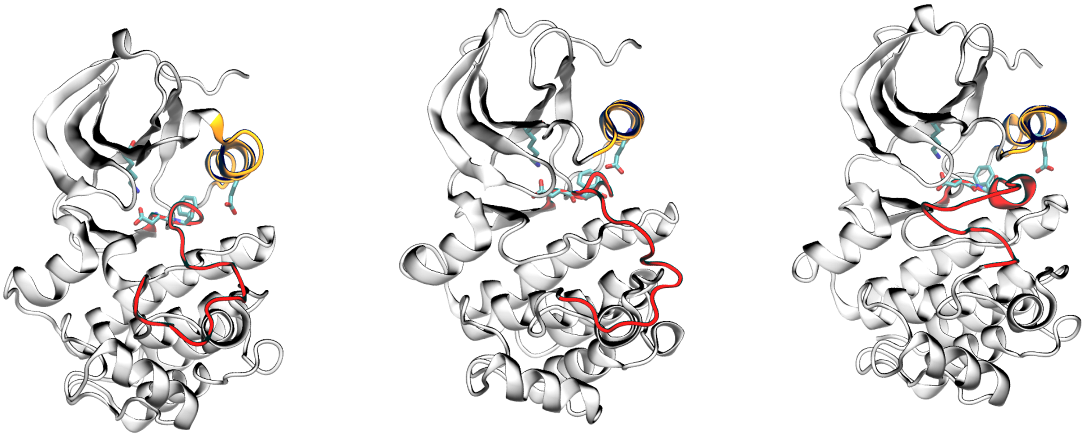

Figure 5: Randomly sampled structures for the intermediate macro state shows large structural heterogenity. While the C-helix is rotated out in all cases, the activation loop can sample a range of different configurations including partially helical structure. The DFG-Asp539 points to the core of the protein.

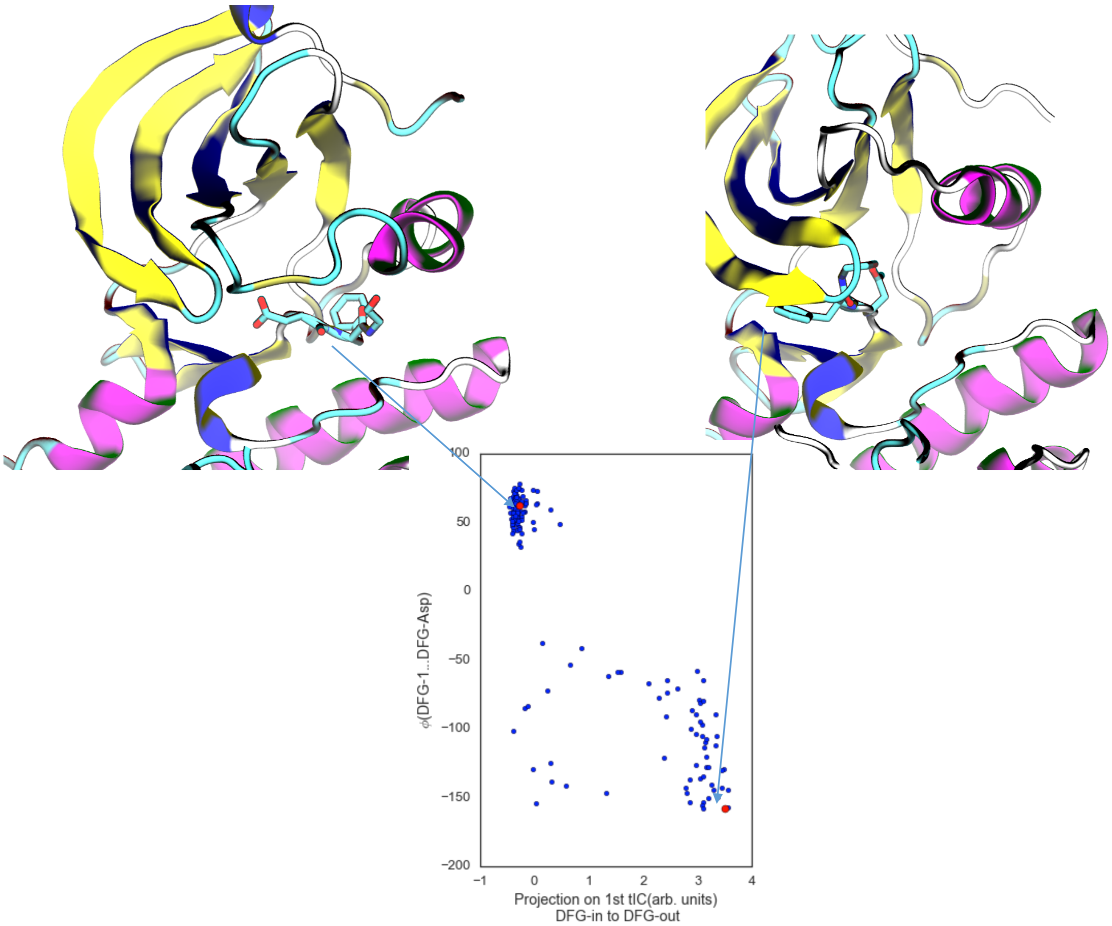

Figure 6: Validation of the tICA model. The first tIC highly correlates with the flipping of the DFG motif to a DFG-out state. The top images show two randmly sampled structures from the min and

max values of the tIC corresponding to the DFG-in and DFG-out states. The bottom graph shows projections of the centroids of the MSM states projected onto their value in the 1st tIC and $\phi_{DFG-ASP}$ showing that the first tIC tracks flipping of the DFG-motif. The most important contacts for the first tIC correspond to the distances between residues Asp539,Phe540 and Ser454, Glu511.

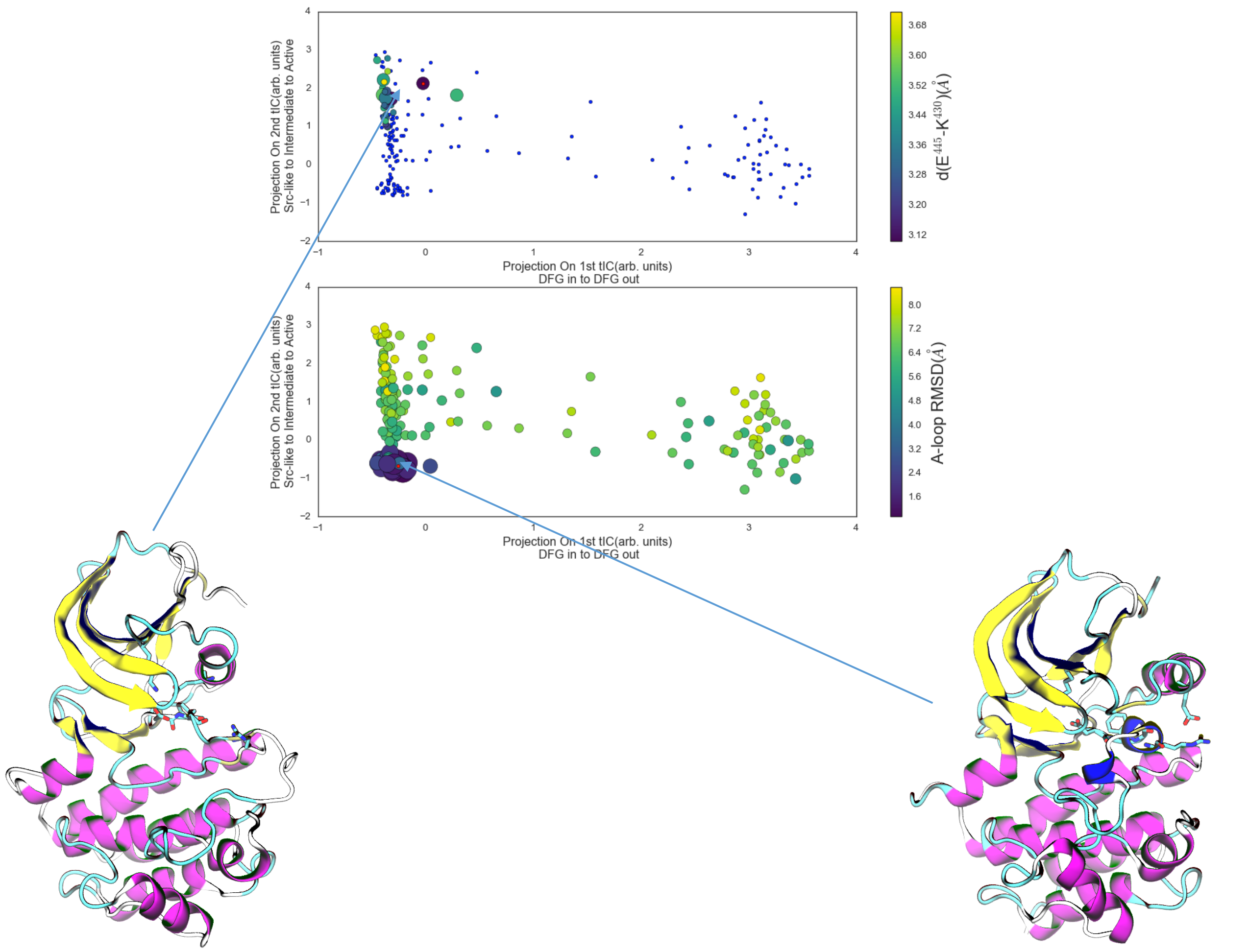

Figure 7: Validation of the tICA model(continued). The 2nd tIC highly correlates with unfolding of the Activation loop and in-ward rotation of the C-helix. The graphs show the projection of the centroids onto the two dominant tICs. The size and color in the top graph corresponds to the distance between $E_{445}^{CD} - K_{430}^{NZ}$ where CD is the delta carbon atom and NZ is the zeta nitrogen. Smaller distance(larger size) indicate that the Glu-Lys bridge is formed and the kinase is in an active state. The size and color in the bottom graph corresponds to the root mean squared deviation(RMSD) of the heavy atoms of the activation loop residues(residues 539-559) from a double helical Src-like state. Smaller RMSD(larger size) indicates a folded A-loop. The two images show the centroid of the state(smaller red dot) from the bottom and top reigon of the 2nd tIC. The 2nd tIC most highly correlates with contacts between Ser543-Val546 , Ser439-Val546, and Glu444-Ser543.

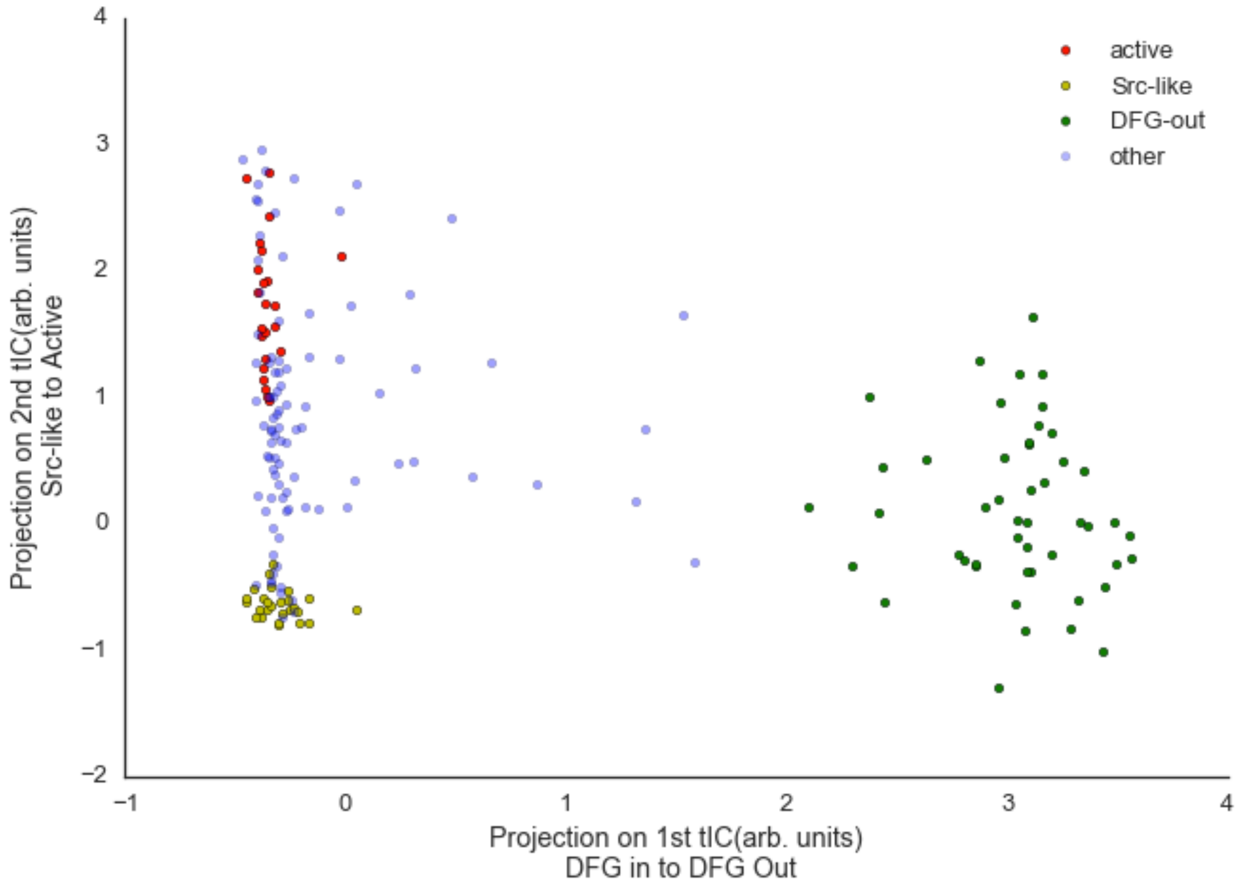

Figure 8: Definiton of the set of active, Src-like like inactive, and DFG-out states used to report the poulation and kinetics. For the active state, we required the distance between $E_{445}^{CD} - K_{430}^{NZ}$ *to be <4 Å 1st tIC value <0, and 2nd tIC value > 2 . For the Src-like states, we picked all states whose centroids had a tIC value <1 in the first dimension, less than 0 in the 2nd dimension, and had an A-loop RMSD to the folded structure be <3 Å. For the DFG out state, we picked all states whose 1st tIC value was higher than 2.*

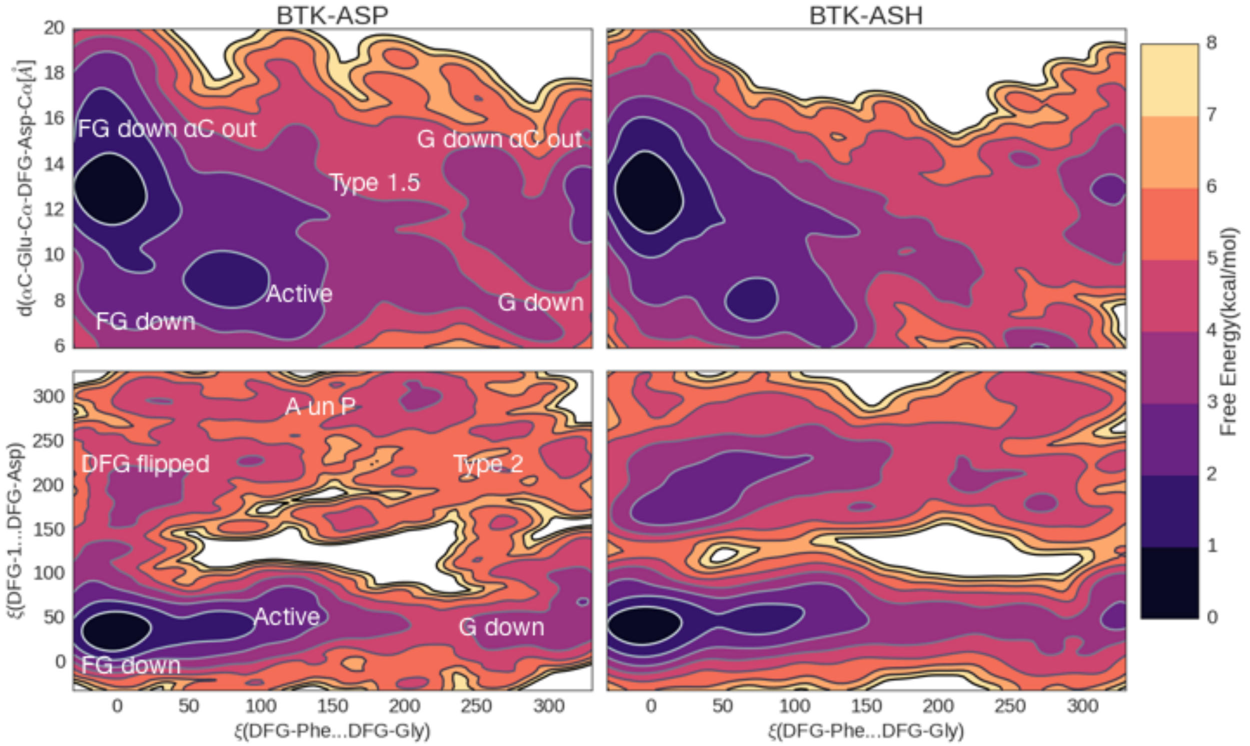

Figure 9: Free-energy projection of the MSM onto the Mobitz order paramters. Upon protonation, the DFG-out state is signifcanlty stabilized. For more details and error bars see Supplementary Figure 12 and Supplemntary Note 1.

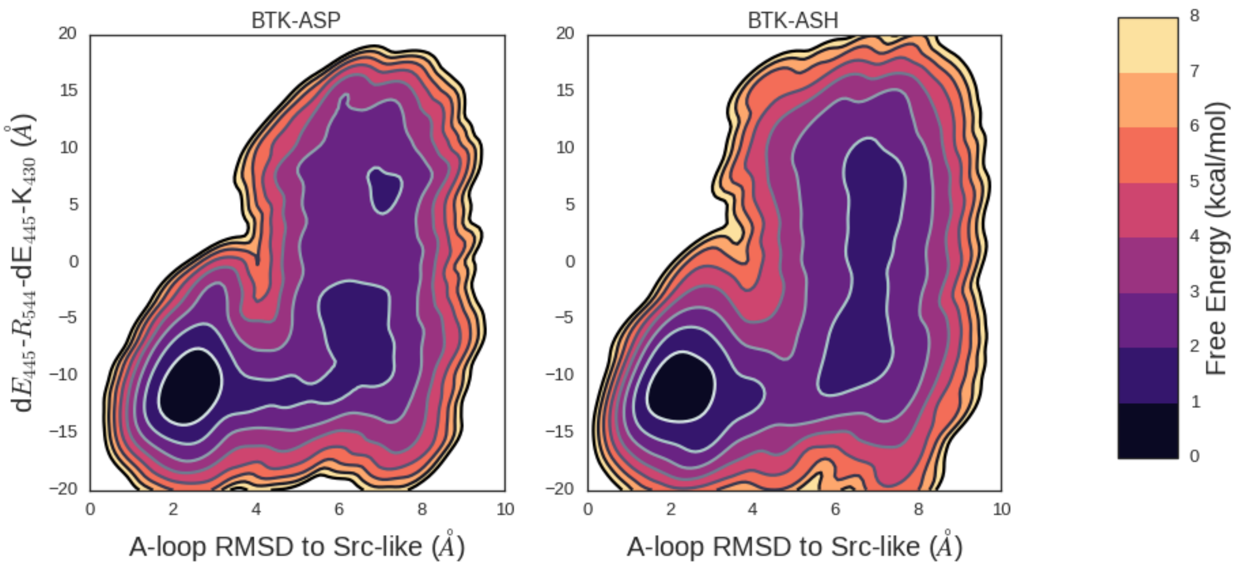

Figure 10: Free-energy projection of the MSM onto two order parameters. The x-axis tracks the hevay atom A-loop RMSD to a double helical Src-like incative state. The y-axis is the difference betweeen the distance meausred for $E_{445}^{CD} - R_{544}^{CZ}$ and $E_{445}^{CD} - K_{430}^{NZ}$. Higher values on the y-axis

indicate inward rotation of the C-helix. This projection completely integrates out the DFG-out state which can both C-helix-in(high value on y-axis) and C-helix-out(low value on y-axis).

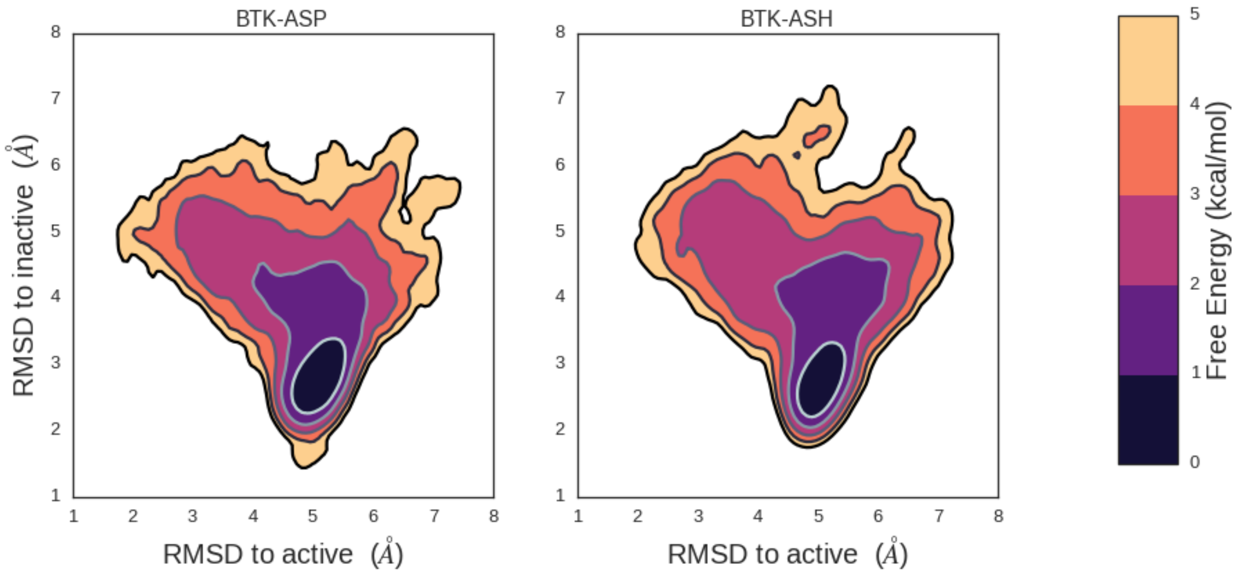

Figure 11: Free-energy projection of the MSM onto two order parameters. The x-axis tracks the all heavy atom to the active state while the y-axis tracks the all heavy atom RMSD to the Src-like structure. Again, this projection sufferes from its inability to separate the DFG-out state. These problems highlight the need for kinetic metircs that are capable of distinguishing structures that are kinetically far apart while being close in RMSD.

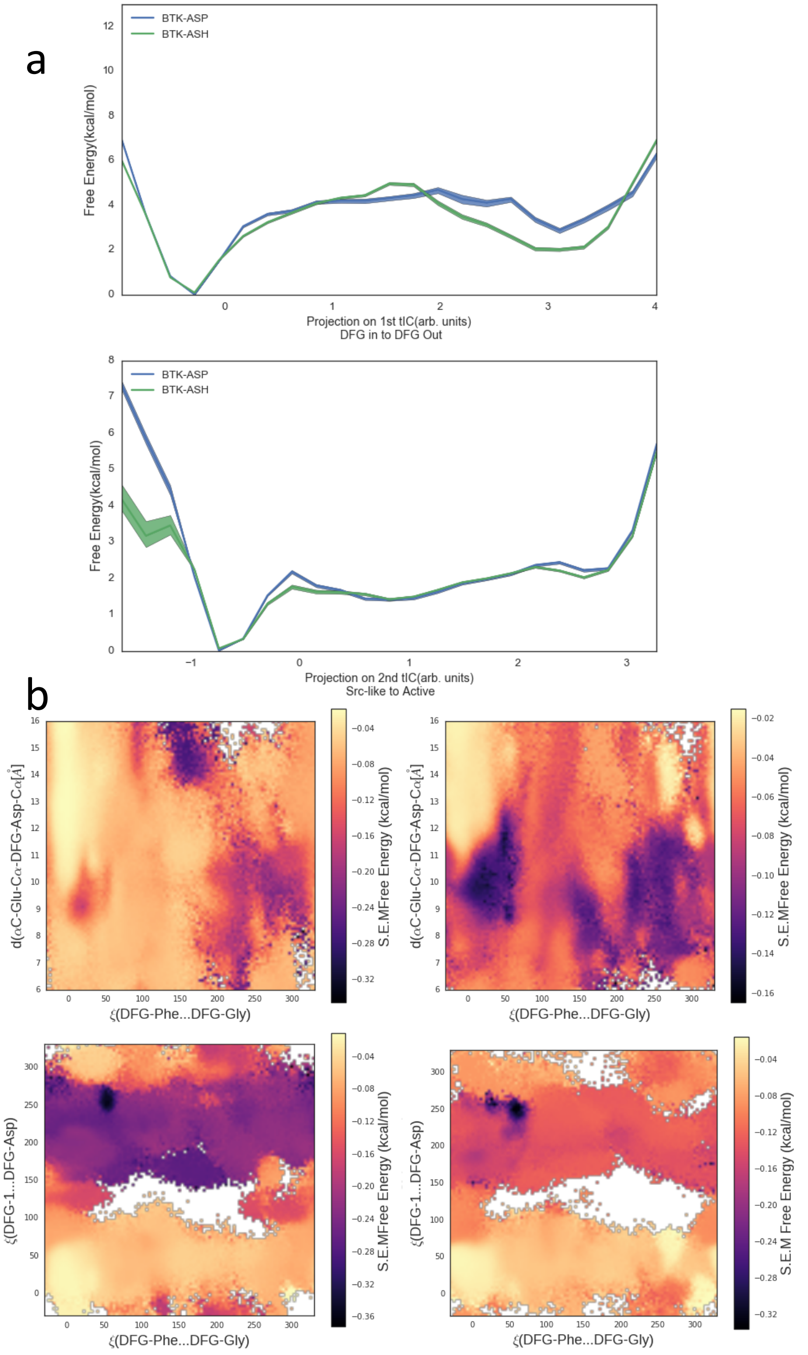

*Figure 12: a) 95% confidence intervals for the thermodynamics of the model projected along the two dominant tICS for the BTK-ASP and BTK-ASH datasets. The error bars were obtained by first emprically estimating the equilibrium population standard deviations using 200 rounds of bootstraping. The standard deviations were then converted into standard error of measurements by dividing it by the squareroot of 200. The 95% confidence interval is 1.96*S.E.M.b). b) Difference in the 95% CI projected along the Mobitz order paramters. This plot was obtained by subtracting the free energy projections generated for upper and lower population bounds.*

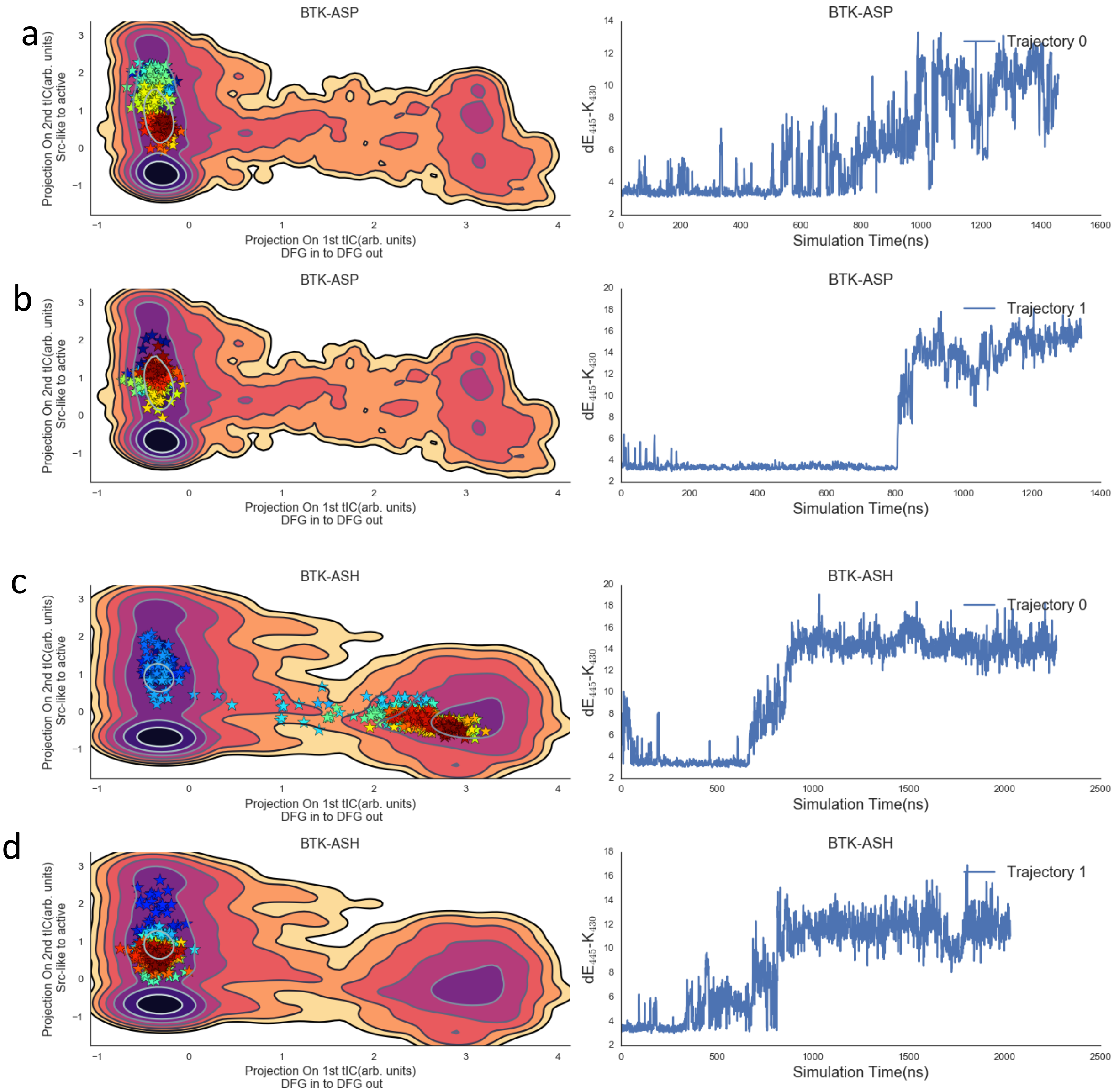

Figure 13: Deactivation doesn't require DFG protonation. For both the BTK-ASP (a & b) and BTK-ASH (c & d), we observed several spontaneous transitions from the C-helix in state to the C-helix out state. The graphs on the left track the trajectories on the tIC free energy surface. The color goes from blue to green to red to indicate simulation time. The graphs on the right track the distance between $E_{445}^{CD} - K_{430}^{NZ}$ *as a function simulation time. Smaller values (<4 Å) indicate C-helix in while larger values indicate C-helix out.*

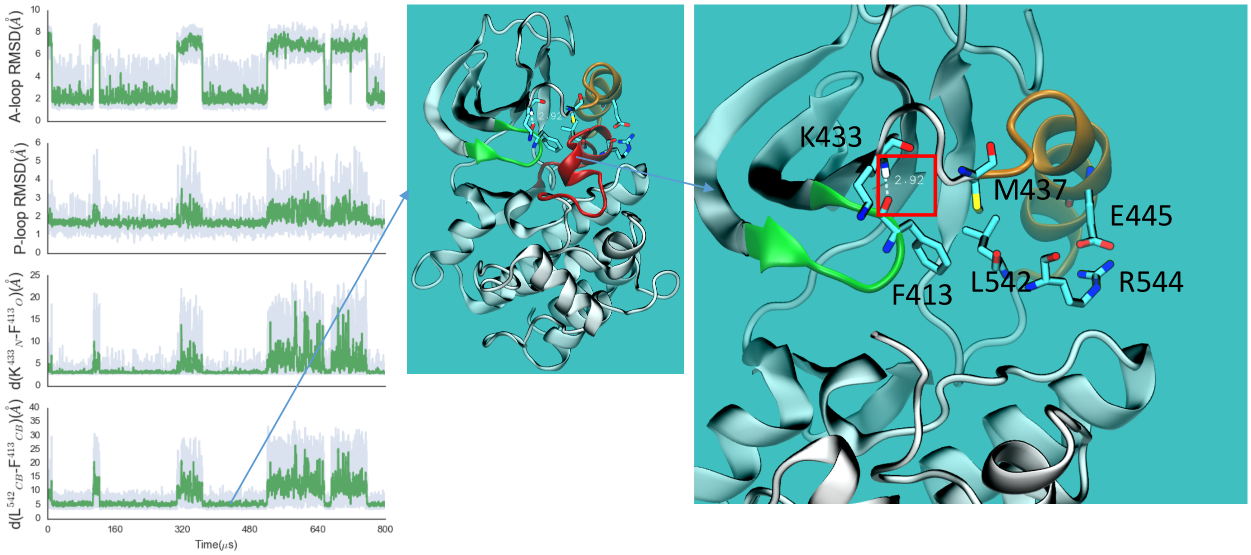

Figure 14:Deactivation to Src-like inactive subdues P-loop dynamics. The left panel shows a MSM MonteCarlo trajectory plotted along several order parameters. Once the model samples the Src-like inactive state (between the 350-450μs mark), the P-loop motions are quenched. This is because of a formation of a hydrogen bond between carbonyl oxygen of the P-loop's Phe413 and the amide group of Lys433, and a hydrophobic patch between F413, L542 and M437. The panels on the right show details of the interactions with the critical residues marked. The activation loop in the last panel is not rendered for the sake of clarity.

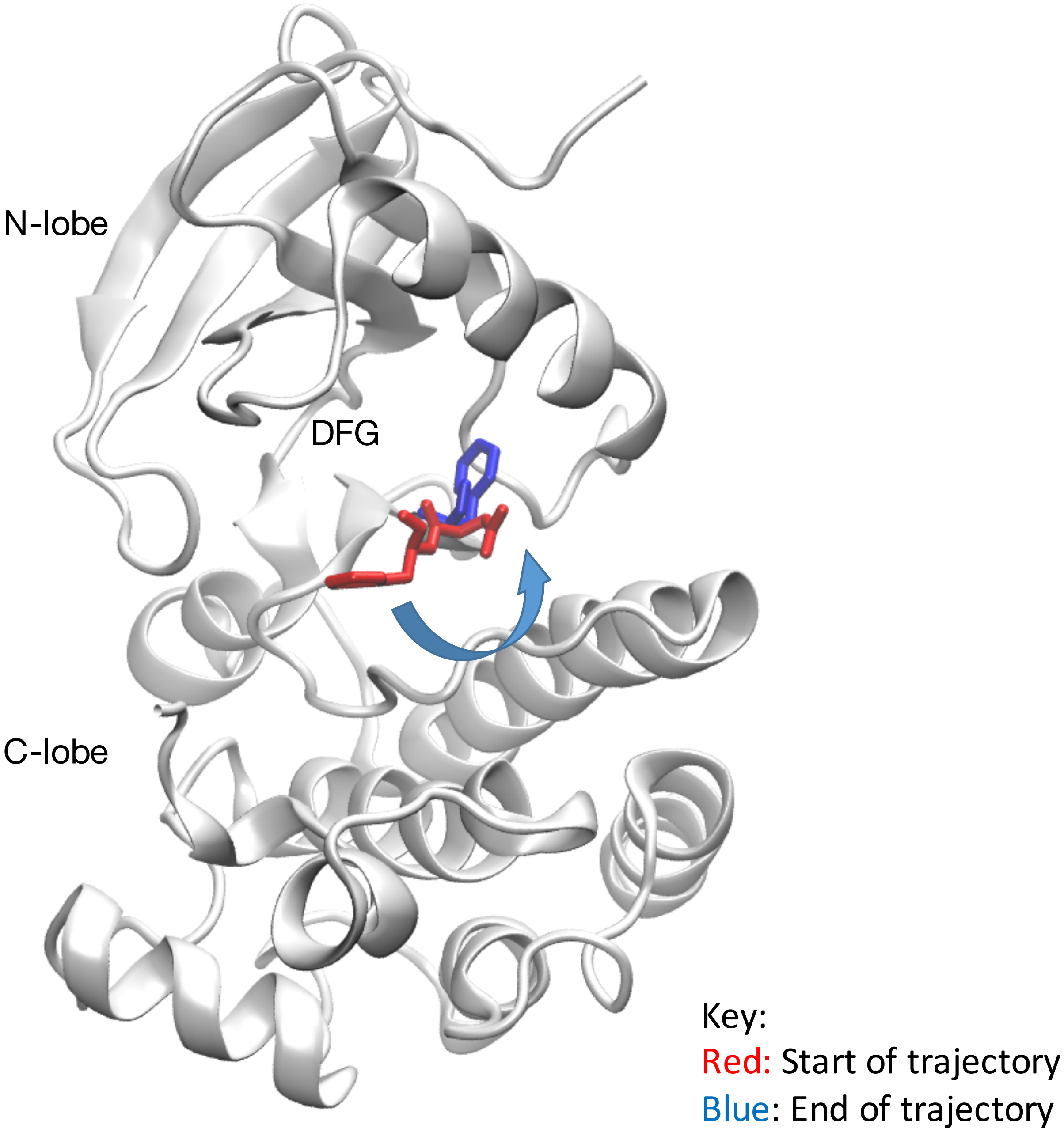

Figure 15: Schematic details of the C-lobe movement. In our DFG flip transitions, the DFG-Phe rotates along the C-lobe (blue arrow) to go from DFG^{out} (red) to DFG^{in} (dark blue).

Supplementary Note 1:
While the Möbitz[30] classification scheme serves as an excellent comparison to our current simulation set, we chose not to explicitly cluster only along those coordinates. Instead we chose to project our trajectories unto several thousand randomly selected contacts and all side chain dihedrals and letting the tICA[13] algorithm identify the slowest coordinates for us. This is done to explicitly reduce the chance of accidently biasing our final MSM. However, our tICA model was able to pick up both the DFG^{in} to DFG^{out} coordinate and C-$helix^{out}$ to C-$helix^{in}$ as the two slowest

transitions(Figure 6-7 above) indicating the ability of MSMs in highlighting interesting and relevant biophysical transitions. Furthermore, our tICA model also identified the unfolding of the activation loop(Figure 7 above) as being highly correlated with activation, a metric that the Möbitz[30] scheme fails to account for. Therefore, all results presented in the paper explicitly use the tICA model with the state definitions outlined in Supplementary Figure 8 (above). However, we provide a classification of the starting pdbs in Table 2 (below) and representative BTK-MD configurations for several of the Möbitz[30] states in Figure 16 (below).

PDB	Möbitz classificaiton
1k2p	active DFG helix C out
3gen	FG down helixC out
3k54	disordered
3ocs	FG down helixC out
3oct	DFG flipped
3p08	other
3pix	AuP MET
3piz	FG down
3pj1	FG down
3pj2	disordered
3pj3	disordered
4nwm	FG down helixC out
4ot5	FG down helixC out
4ot6	FG down helixC out
4otq	FG down helixC out
4otr	FG down helixC out
4xi2	FG down helixC out
4rfy	FG down helixC out
4y93	FG down helixC out
4rfz	FG down helixC out
4otf	FG down helixC out
4rg0	FG down helixC out
4y95	disordered

Table 3: Möbitz classification of the un minimized non-mutated raw starting structures. All values were obtained using the classification scheme provided in the original paper[30].

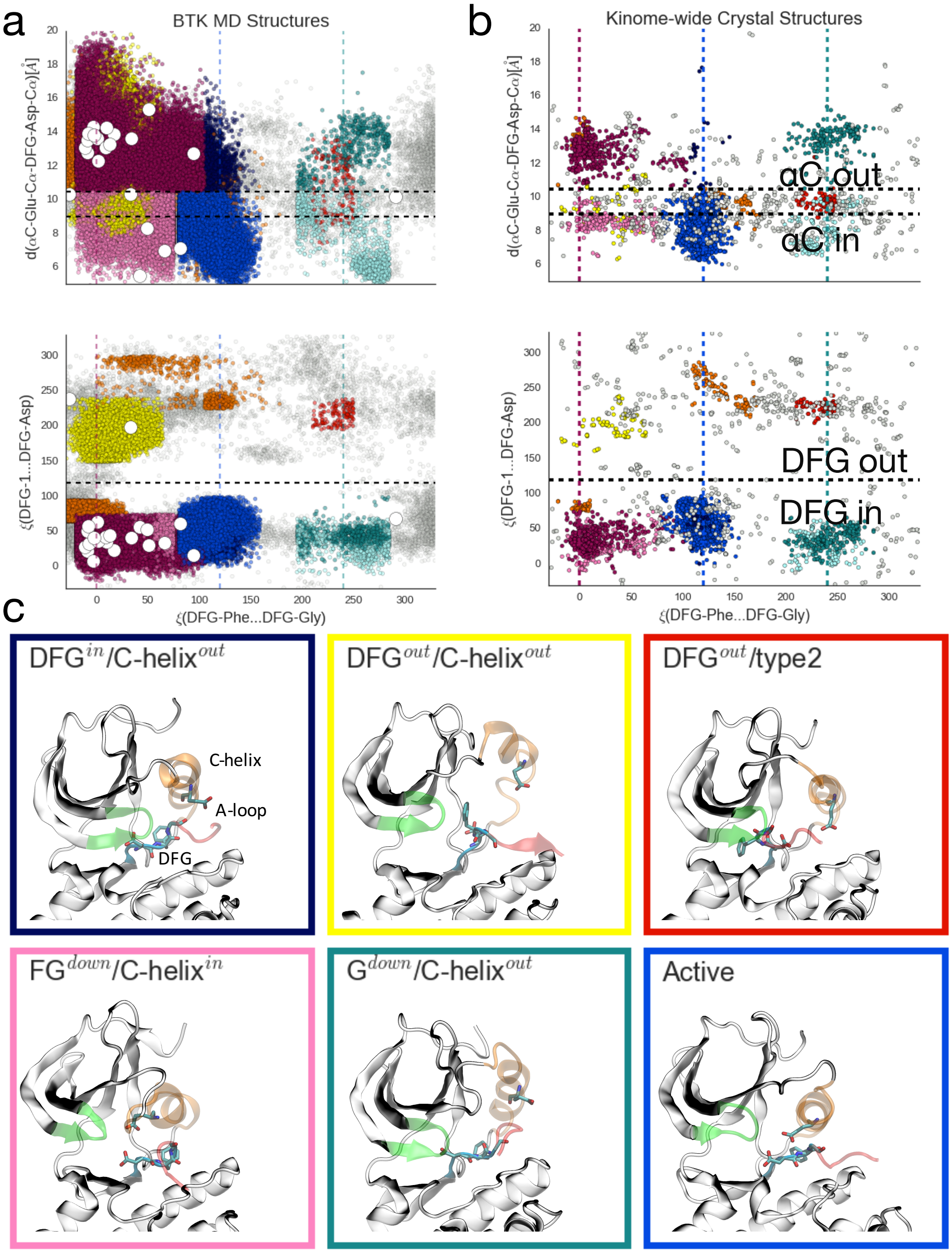

Figure 16: BTK's apo domain contains kinome-wide conformational plasticity. Comparison of 9% of MD generated structures (a) against all known publically available kinase domain structures (b) projected along three key degrees of freedom as outlined in Möbitz et al[30]. We used the data and classification scheme provided in [30] to generate (b). The top y-coordinate tracks the C-helix^{in} to C-helix^{out} transition while the bottom y-coordinate tracks the DFG^{in} to DFG^{out} transition. The common x-axis subdivides the conformations into pharmacologically relevant states of the DFG motif. The white circles in (a) correspond to the starting configurations for the

MD simulations. The points are colored according to their Möbitz cluster: active (blue), DFG^{in}-αC-helix^{out} (dark blue), FG-down (pink), FG-down –αC-helix^{out} (dark magenta), G-down (light cyan), G-down-αC-helix^{out} (dark cyan), DFG^{out} (yellow), DFG^{out} type 2 (red), four different variants of A-loop under P-loop (orange), and disordered/other (grey). C). Randomly sampled structures for six of the kinase classes are shown with box colors identifying the corresponding Möbitz classification. We have hidden the C-terminal end of the A-loop (red) for the sake of clarity.

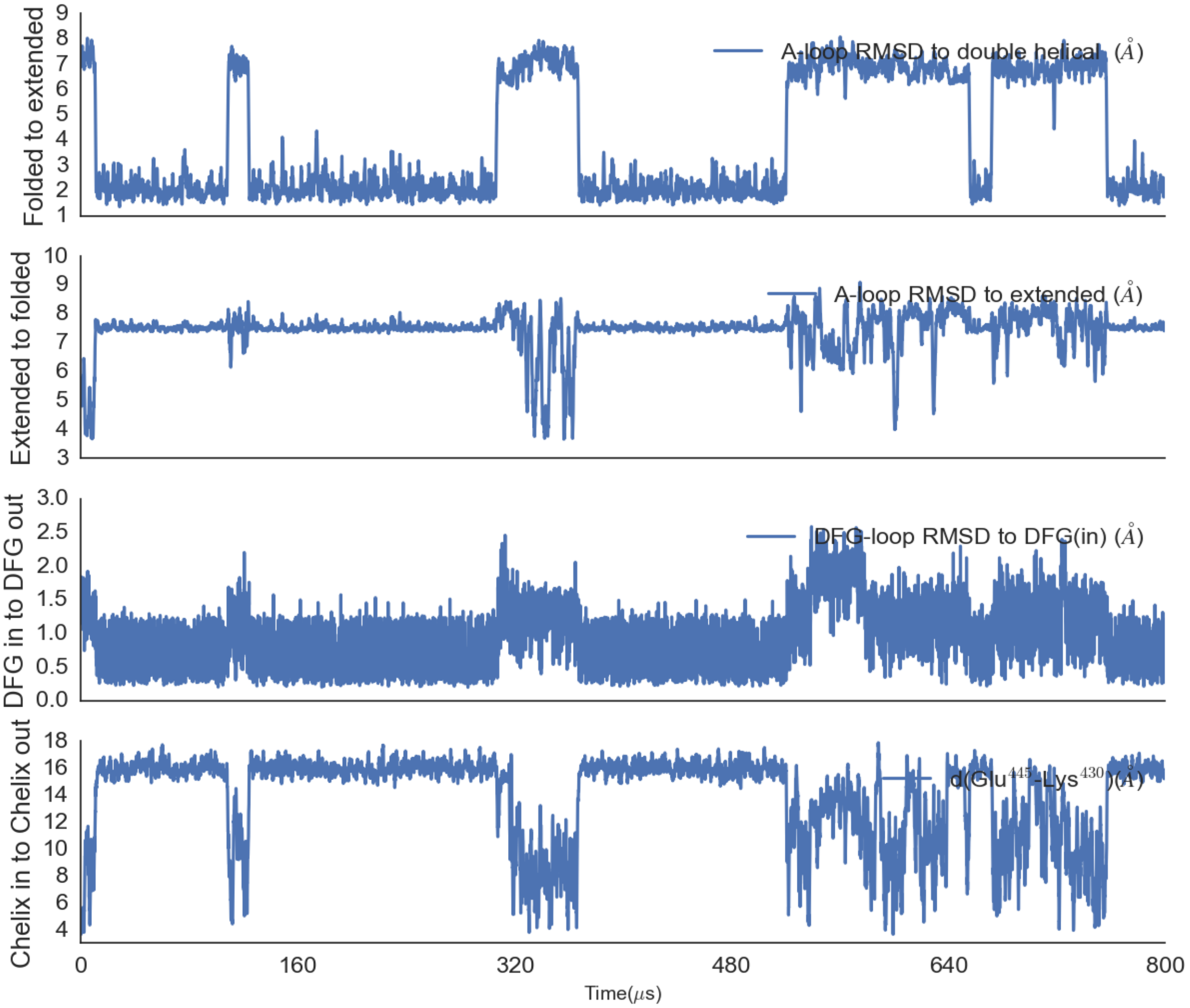

Figure 17: A-loop RMSD during and after DFG flip is different from either double helical or extended. Projection of the Monte Carlo trajectory from the main paper along 4 order parameters. The A-loop RMSD (top two panels) upon DFG flip (3rd panel 480 microseconds onwards) is different from either the Src-like folded state or the extended state. The traces correspond to moving averages across 10 frames.

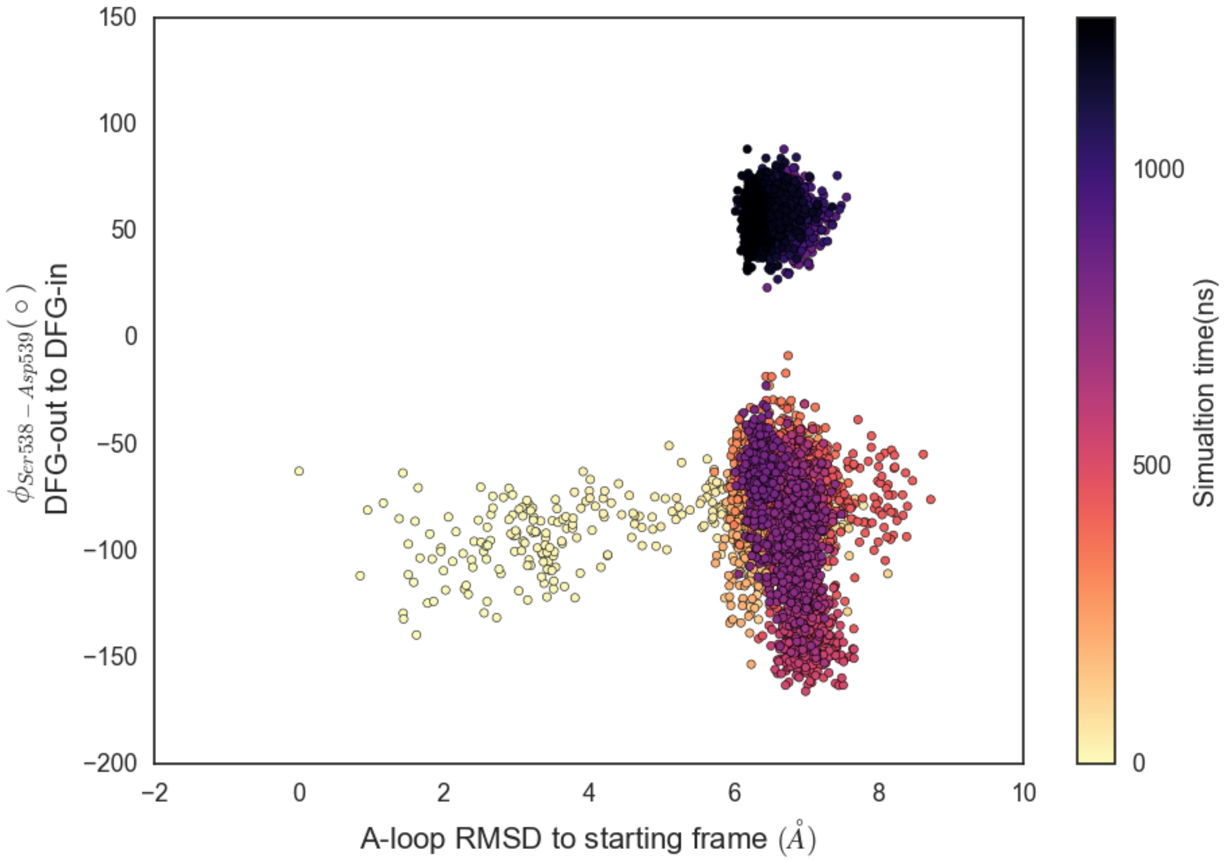

Figure 18: DFG-flip occurs after a large scale conformational change in the A-loop. Projection of the trajectory shown in Figure 5 of the main text as a function of the A-loop RMSD and simulation time.

Supporting Note 2:
DFG-ASP539 undergoes a large-scale conformational change from a mostly hydrophilic environment to a mostly hydrophobic environment (Figure 19 below) when going from the DFG^{in} to the DFG^{out} state. Previous experimental and computational[31,32] work suggests that the DFG-Aspartate residue might be protonated in the DFG^{out} state to reduce the free-energy cost of burying a charged residue inside the protein's core.
To ascertain the likelihood of ASP539 protonation, we used the H++ server[33] to calculate the pKa of DFG-ASP539 in the only reported DFG^{out} crystal structure [34] 3PJ3. To that end, we removed the protein (heavy atom only) from 3PJ3 and built the missing residues using Modeller. Based upon the choice of the internal dielectric, the web service estimated the pKa from 5.9 (internal dielectric 10, external dielectric 80, pH 7.2) to 9.6 (internal dielectric 4, external dielectric 80, pH 7). This is several pH units away from the reported solution pKa of ~4, indicating ASP539's proclivity for being protonated in the DFG^{out} state. Other titratable residues such as His519 or Asp521 had pKa<4 in several differing starting configurations indicating that the residues are unlikely to protonate at neutral pH of 7.2. We therefore left those residues in their de-protonated states. This is not too surprising because the local environment around these residues is mostly solvent exposed in both the DFG^{in} and DFG^{out} states.

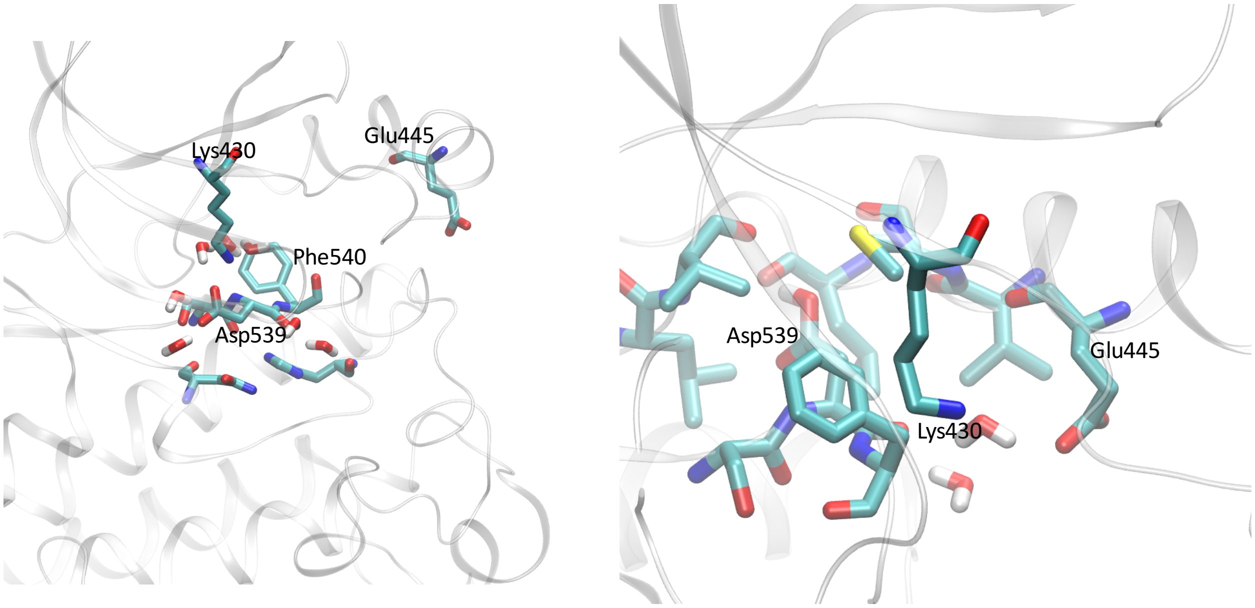

Figure 19: DFG-Asp539 goes from a solvent exposed environment to mostly hydrophobic environment upon DFG flip. Left, DFG-Asp539 is solvated by several water molecules in the Src-like inactive DFG^{in} state. Right, protonated DFG-Asp539 is surrounded by several hydrophobic valine and methionine residues. In both cases, we used VMD's atom selection language to find all water and protein residues within 3 A of Asp539.

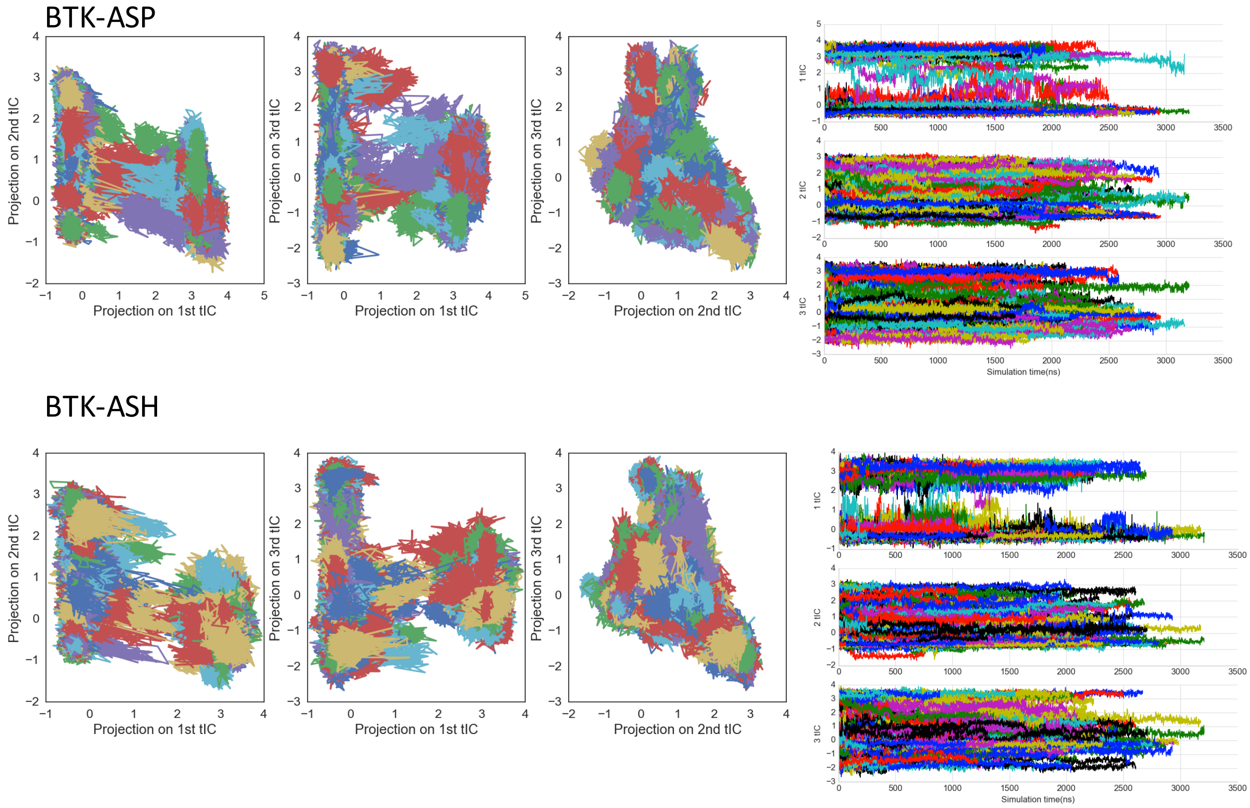

Figure 20: Projection of 1.8ms (20% of data obtained by subsampling to 1ns) of BTK-ASP and BTK-ASH datasets unto three dominant tICs, shows that we have significant number of partial and complete crossovers along each coordinate. Note that the MSM method doesn't require any single trajectory to completely cross over a coordinate but can use the information from partial crossovers to gain global equilibrium statistics. Each color corresponds to a different trajectory. The panels on the right trace the trajectories as a function of simulation time.

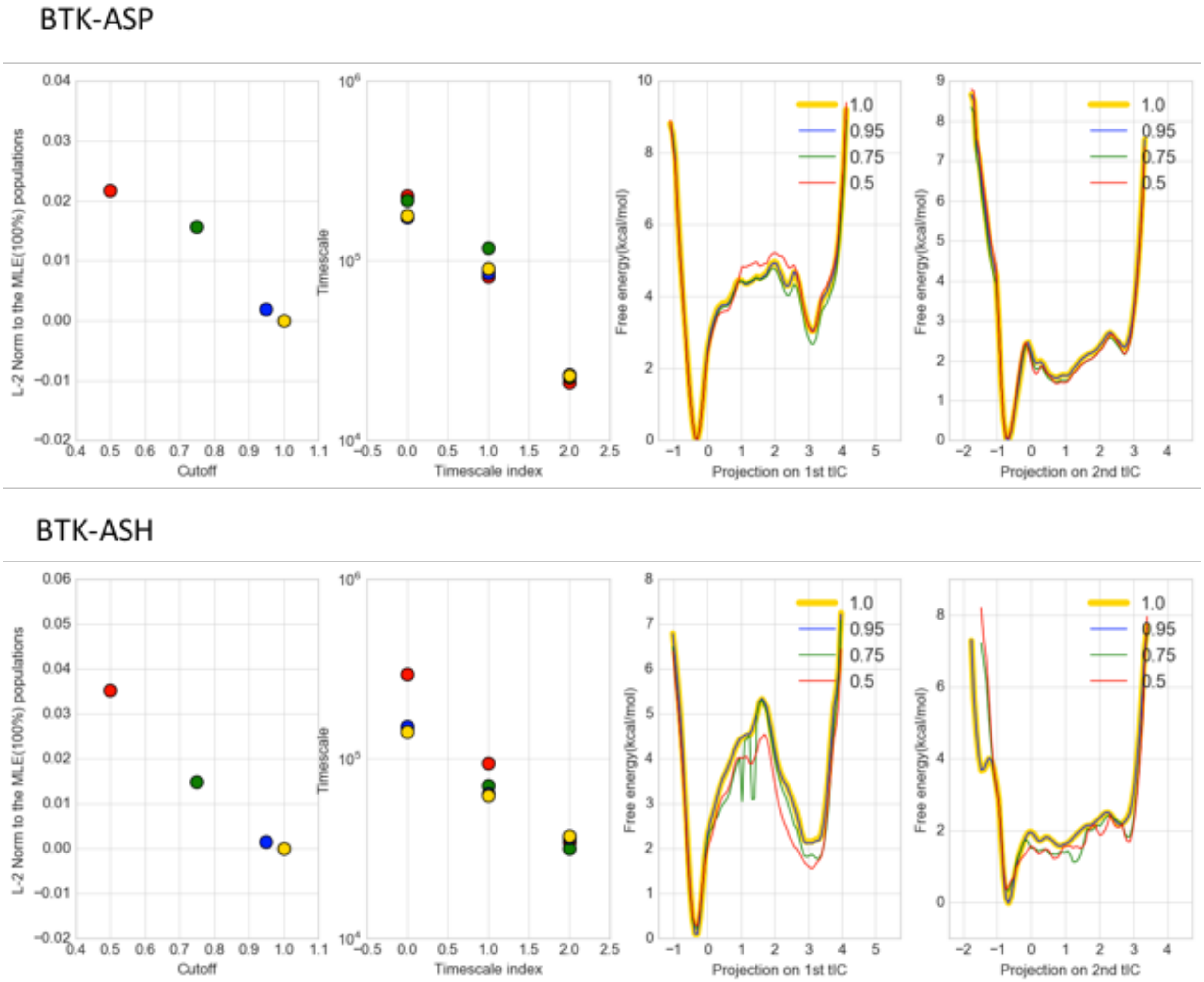

Figure 21: Convergence of MSMs as a function of trajectory length shows that the MSM thermodynamics and kinetics are robust. For both ensembles, we restricted each individual trajectory's state assignments from 50 to 95% of the its final length. We next re-computed the MSM for the sub-sampled data and compared the population (via L-2 norm), longest timescales, and projections along dominant tICs to the complete data solution(gold). As it can be seen, our models converge to within 0.5kcal/mol of their final estimates even at 50% of the overall data.

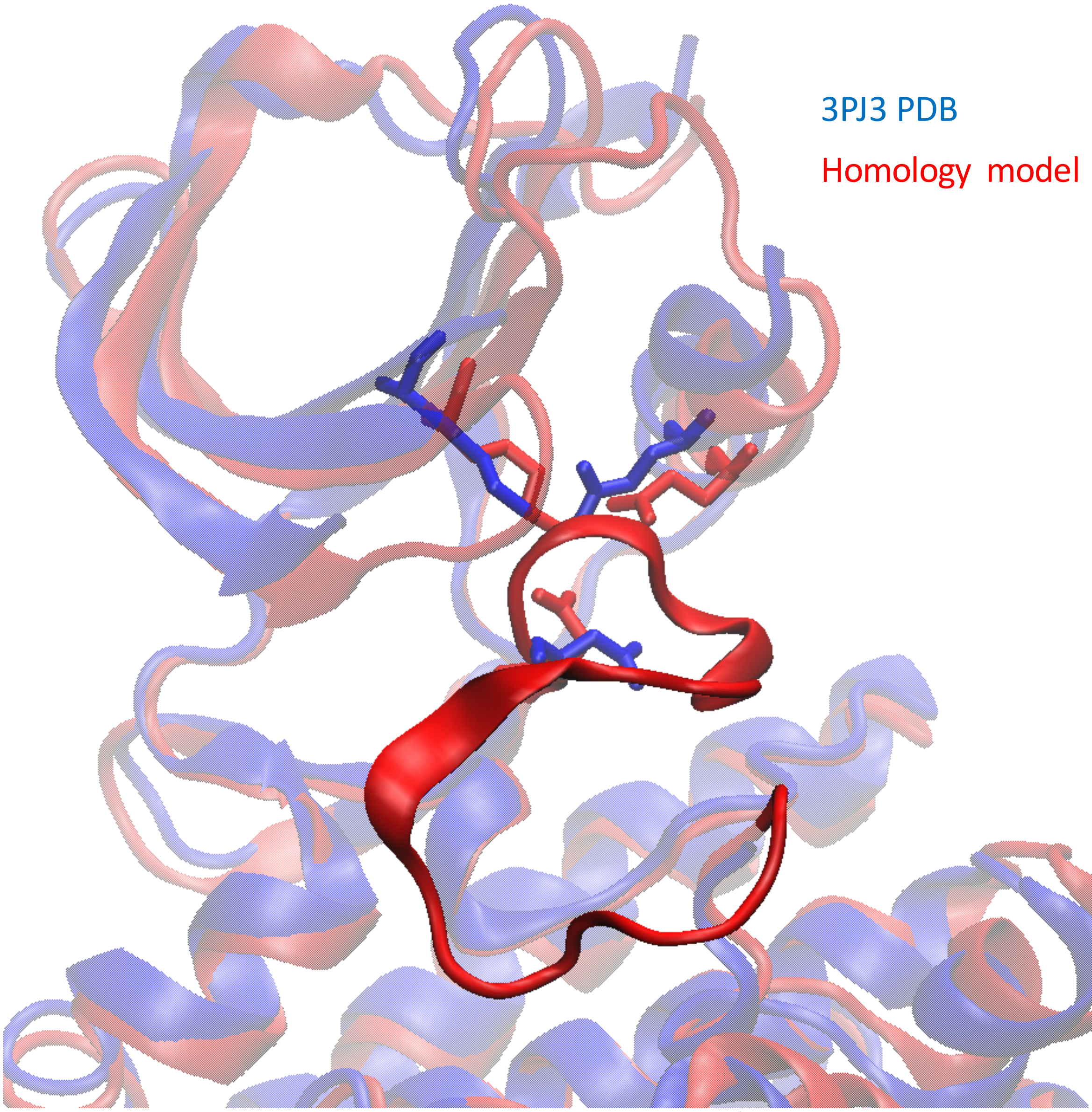

Figure 22: The starting A-loop configuration for the DFG^{out} state. The blue model corresponds the PDB coordinates from 3PJ3 while the red model corresponds to the energy minimized model generated via the Modeller software. The A-loop (red, opaque was built as an extended chain).

1. Berman, H. M. The Protein Data Bank. *Nucleic Acids Res.* **28,** 235–242 (2000).
2. Eswar, N. *et al.* Comparative protein structure modeling using MODELLER. *Curr. Protoc. Protein Sci.* **Chapter 2,** Unit 2.9 (2007).
3. Salomon-Ferrer, R., Case, D. A. & Walker, R. C. An overview of the Amber biomolecular simulation package. *Wiley Interdiscip. Rev. Comput. Mol. Sci.* **3,** 198–210 (2013).
4. Götz, A. W. *et al.* Routine Microsecond Molecular Dynamics Simulations with AMBER on GPUs. 1. Generalized Born. *J. Chem. Theory Comput.* **8,** 1542–1555 (2012).
5. Salomon-Ferrer, R., Götz, A. W., Poole, D., Le Grand, S. & Walker, R. C. Routine Microsecond Molecular Dynamics Simulations with AMBER on GPUs. 2. Explicit

Solvent Particle Mesh Ewald. *J. Chem. Theory Comput.* **9,** 3878–3888 (2013).
6. Lindorff-Larsen, K. *et al.* Improved side-chain torsion potentials for the Amber ff99SB protein force field. *Proteins* **78,** 1950–8 (2010).
7. Jorgensen, W. L., Chandrasekhar, J., Madura, J. D., Impey, R. W. & Klein, M. L. Comparison of simple potential functions for simulating liquid water. *J. Chem. Phys.* **79,** 926 (1983).
8. Eastman, P. *et al.* OpenMM 4: A Reusable, Extensible, Hardware Independent Library for High Performance Molecular Simulation. *J. Chem. Theory Comput.* **9,** 461–469 (2013).
9. Darden, T., York, D. & Pedersen, L. Particle mesh Ewald: An N · log(N) method for Ewald sums in large systems. *J. Chem. Phys.* **98,** 10089 (1993).
10. Shirts, M. & Pande, V. S. Screen Savers of the World Unite! *Science* **290,** 1903–1904 (2000).
11. Hess, B., Bekker, H., Berendsen, H. J. C. & Fraaije, J. G. E. M. LINCS: A linear constraint solver for molecular simulations. *J. Comput. Chem.* **18,** 1463–1472 (1997).
12. Prinz, J.-H. *et al.* Markov models of molecular kinetics: generation and validation. *J. Chem. Phys.* **134,** 174105 (2011).
13. Schwantes, C. R. & Pande, V. S. Improvements in Markov State Model Construction Reveal Many Non-Native Interactions in the Folding of NTL9. *J. Chem. Theory Comput.* **9,** 2000–2009 (2013).
14. Shukla, D., Meng, Y., Roux, B. & Pande, V. S. Activation pathway of Src kinase reveals intermediate states as targets for drug design. *Nat. Commun.* **5,** 3397 (2014).
15. Pérez-Hernández, G. *et al.* Identification of slow molecular order parameters for Markov model construction. *J. Chem. Phys.* **139,** 15102 (2013).
16. Kohlhoff, K. J. *et al.* Cloud-based simulations on Google Exacycle reveal ligand modulation of GPCR activation pathways. *Nat. Chem.* **6,** 15–21 (2014).
17. McGibbon, R. T. & Pande, V. S. Variational cross-validation of slow dynamical modes in molecular kinetics. *J. Chem. phyics* **142,** (2015).
18. Nüske, F., Keller, B. G., Pérez-Hernández, G., Mey, A. S. J. S. & Noé, F. Variational Approach to Molecular Kinetics. *J. Chem. Theory Comput.* **10,** 1739–52 (2014).
19. Noé, F. & Clementi, C. Kinetic distance and kinetic maps from molecular dynamics simulation. *J. Chem. Theory Comput.* **11,** 5002–5011 (2015).
20. McGibbon, R. T. & Pande, V. S. Identification of simple reaction coordinates from complex dynamics. 1–16 (2016).
21. Mcgibbon, R. T. *et al.* MDTraj: a modern, open library for the analysis of molecular dynamics trajectories. *bioRxiv* 9–10 (2014).
22. Beauchamp, K. A. *et al.* MSMBuilder2: modeling conformational dynamics on the picosecond to millisecond scale. *J. Chem. Theory Comput.* **7,** 3412–3419 (2011).
23. Pérez, F. & Granger, B. E. IPython: a System for Interactive Scientific Computing. *Comput. Sci. Eng.* **9,** 21–29 (2007).
24. Hunter, J. D. Matplotlib: A 2D graphic environment. *Comput. Sci. Eng.* **9,** 90–95 (2007).
25. Pedregosa, F. & Varoquaux, G. Scikit-learn: Machine learning in Python. *J. Mach. Learn.* **12,** 2825–2830 (2011).
26. Humphrey, W., Dalke, A. & Schulten, K. VMD: visual molecular dynamics. *J. Mol. Graph.* **14,** 33–8, 27–8 (1996).
27. Amitabh Varshney, Frederick P. Brooks, Jr., Jr. William, W. V. W. Linearly Scalable Computation of Smooth Molecular Surfaces. *IEEE Comput. Graph. Appl.* **14,** (1994).

28. Frishman, D. & Argos, P. Knowledge-based protein secondary structure assignment. *Proteins* **23,** 566–79 (1995).
29. Sultan, M. M., Kiss, G., Shukla, D. & Pande, V. S. Automatic Selection of Order Parameters in the Analysis of Large Scale Molecular Dynamics Simulations. *J. Chem. Theory Comput.* **10,** 5217–5223 (2014).
30. Möbitz, H. The ABC of protein kinase conformations. *Biochim. Biophys. Acta - Proteins Proteomics* **1854,** 1555–1566 (2015).
31. Shan, Y. *et al.* A conserved protonation-dependent switch controls drug binding in the Abl kinase. *Proc. Natl. Acad. Sci. U. S. A.* **106,** 139–44 (2009).
32. Shan, Y., Arkhipov, A., Kim, E. T., Pan, A. C. & Shaw, D. E. Transitions to catalytically inactive conformations in EGFR kinase. *Proc. Natl. Acad. Sci. U. S. A.* **110,** 7270–5 (2013).
33. Gordon, J. C. *et al.* H++: a server for estimating pKas and adding missing hydrogens to macromolecules. *Nucleic Acids Res.* **33,** W368-71 (2005).
34. Kuglstatter, A. *et al.* Insights into the conformational flexibility of Bruton's tyrosine kinase from multiple ligand complex structures. *Protein Sci.* **20,** 428–436 (2011).
